# Information theory and diagnostic reasoning in primary care: a framework for navigating uncertainty

**DOI:** 10.1093/fampra/cmag083

**Published:** 2026-10-16

**Authors:** Brian H Willis

**Affiliations:** School of Health Sciences, University of Birmingham, Edgbaston, Birmingham B15 2TT, United Kingdom

**Keywords:** information theory, diagnostic reasoning, primary health care, clinical decision-Making

## Abstract

This is an overview of how information theory offers a rigorous and intuitive framework for understanding diagnostic reasoning in primary care. Primary care clinicians frequently encounter patients early in their illness trajectory, when symptoms are vague, evolving, or non-specific, creating high diagnostic uncertainty. Information theory, through its core concepts of entropy, information content, and information gain, provides a structured way to quantify this uncertainty and understand how it changes as new clinical information is gathered. Entropy reflects the uncertainty associated with the breadth of the differential diagnosis; information content captures how surprising or discriminating a finding is; and information gain describes how much a particular question, sign, or test reduces uncertainty.

Applied to the consultation, these principles explain why rare but specific features are disproportionately informative, why sequences of low-yield tests may add little value, and how targeted history-taking can outperform broad investigation. The framework complements Bayesian reasoning, hypothetico-deductive approaches, and intuitive pattern recognition by clarifying the informational value that underpins each. Through clinical examples, the paper illustrates how information theory sharpens diagnostic strategy, supports efficient use of investigations, and helps clinicians navigate uncertainty with greater clarity and safety. Ultimately, it positions diagnosis as a scientific process of uncertainty reduction central to primary care practice.

Key messages

- Information theory provides a framework for diagnostic uncertainty.
- Entropy quantifies uncertainty across the differential diagnosis.
- Information gain explains the value of clinical findings and tests.
- The framework complements Bayesian and intuitive reasoning.
- It supports more structured diagnostic decision-making.

## Introduction

Diagnostic reasoning forms a central part of everyday general practice. Patients often present early in their illness, with symptoms that are evolving or nonspecific [1]. Unlike settings where definitive investigations are immediately available, primary care requires clinicians to make sense of uncertainty while symptoms are still developing and before a clear diagnostic pattern has emerged [2]. The task is therefore to work safely and effectively under uncertainty while progressively identifying the underlying problem.

Information theory, originally developed by Claude Shannon in the 1940s to understand communication systems [3], offers a powerful framework for thinking about diagnostic uncertainty [4–6]. It provides a mathematical way of quantifying how unsure we are, how much new data can help, and how our diagnostic understanding improves as uncertainty reduces. Whilst the language of information theory, which includes terms such as *entropy*, *information content*, and *information gain*, may appear esoteric, the underlying insights are intuitive and highly relevant to primary care.

In this article, how information theory illuminates fundamental aspects of diagnostic reasoning, particularly under uncertainty, will be explored. These principles are then applied to clinical case examples, illustrating their practical value to everyday primary care. As such, this article does not propose a new model of diagnostic reasoning but rather shows how information theory provides a unifying conceptual framework for understanding how diagnostic uncertainty changes during the consultation.

## Diagnosis as a problem of reducing uncertainty

Diagnosis begins with eliciting information [7]. As the General Practitioner (GP) listens to the patient's story and gathers early details, a range of possible diagnoses begins to take shape. A symptom such as chest pain, fatigue or abdominal discomfort rarely indicates a single condition at the outset; rather, it prompts the clinician to consider several possibilities that will be refined as more information becomes available. Each subsequent step in the consultation that includes taking a history, examining the patient and selecting investigations serves to refine these possibilities further, gradually narrowing the field until one or more diagnoses become sufficiently probable to guide reasonably safe and appropriate action [8].

Information theory gives us a way of describing this uncertainty precisely. Instead of treating the differential diagnosis as a vague list, we can think of it as a probability distribution: each potential diagnosis carries some probability based on prevalence, clinical context, and prior knowledge. As we gather more information, this distribution changes shape where some probabilities rise, others fall, and the overall uncertainty decreases.

Crucially, this provides a way of thinking not only about which diagnosis is most likely, but how much uncertainty remains, and how valuable a particular question, examination finding, or test might be in moving us towards clarity.

## Principles of information theory in diagnostic reasoning

There are three core principles of information theory that are particularly relevant to diagnostic reasoning: *entropy*, *information content,* and *information gain* [4–6, 9].

### Entropy

Similar to its use in physics, in information theory, entropy describes how unpredictable or uncertain a situation is. In diagnostic terms, entropy is high when several diagnoses appear similarly plausible and low when one diagnosis dominates. Entropy is measured in bits and is calculated using a logarithmic scale (see the Appendix for the mathematical formulation). For example, suppose a patient presents with nonspecific symptoms and the GP initially considers four diagnoses {A, B, C, D} as equally likely (each with probability 0.25). The probability distribution is therefore *P* = (0.25, 0.25, 0.25, 0.25). Each diagnosis contributes equally to the total entropy, with each contributing 0.5 bits, giving a total entropy of *H* = 2.0 bits.

When there is no uncertainty and one diagnosis dominates with certainty, *H* = *0*, but as the plausible diagnoses increase, the uncertainty, i.e. the entropy, *H*, increases.

Thus, a patient presenting with isolated fatigue in primary care has high diagnostic entropy. Similarly, symptoms such as back pain, dizziness, or dyspepsia all generate broad differentials and the entropy is high. In contrast, a feature that is highly correlated with one diagnosis and rarely occurs in other diagnoses is discriminatory and may cause the entropy to fall rapidly when present. For example, a patient presenting with sudden-onset left-sided weakness suggestive of a stroke would have far lower entropy.

Furthermore, entropy decreases as informative findings accumulate, although common or low-yield findings may have little impact on it. This leads us to the importance of information content.

### Information content

Essentially, information content (also known as the *surprisal*) captures how surprising or unexpected a finding is [4, 9]. Unlike entropy, which quantifies the uncertainty across the entire probability distribution of possible diagnoses, information content quantifies the surprise associated with a single clinical finding. So if entropy measures overall uncertainty, information content measures how much a single finding surprises you and therefore how much it forces you to update your diagnostic thinking. Like entropy, it is measured in bits.

To put into context, a feature which is completely expected carries 0 bits of information, but observing a rare feature that has a probability of 1% carries 6.6 bits of information.

Thus, the more unexpected the finding, the more information it carries. In diagnostic reasoning, a patient presenting with a common symptom seen in a lot of patients provides very little new information about the distribution of possible diagnoses. In contrast, rare findings are far more informative. Rare features such as digital clubbing, a malar rash, or a non-blanching petechial rash carry more information and can dramatically narrow the differential diagnosis.

Furthermore, information content is context-dependent, and this helps explain why some tests are more helpful than others. Thus, a test with a high sensitivity and specificity may still have low information content if the result was highly expected.

It is also important not to confuse clinical severity with informational value. For example, fever in a child might be alarming to a parent, but it carries very low information content because it is extremely common across many benign conditions. Conversely, a very subtle but rare sign such as Koplik spots may have very high information content because it strongly narrows the differential diagnosis.

In summary, the information content reflects how much a clinician must change their thinking on the differential diagnosis.

### Information gain

Information gain quantifies how much a new piece of data reduces uncertainty. In information theory, this uncertainty is measured by entropy, so information gain is simply the difference between the entropy before we obtain new information and the entropy after we obtain it.

Consider our earlier example where, before observing any features, the GP considered four possible diagnoses, {A, B, C, D}, equally likely, so *P* = *(0.25, 0.25, 0.25, 0.25)* and the entropy, $H_{before}=2\;bits$. Suppose the GP elicits a new clinical finding, such as exertional chest pain, which shifts the probability strongly towards one diagnosis, giving *P* = *(0.70, 0.10, 0.10, 0.10).* The resulting change in the probability distribution is illustrated in Fig. 1.

**Figure 1:**
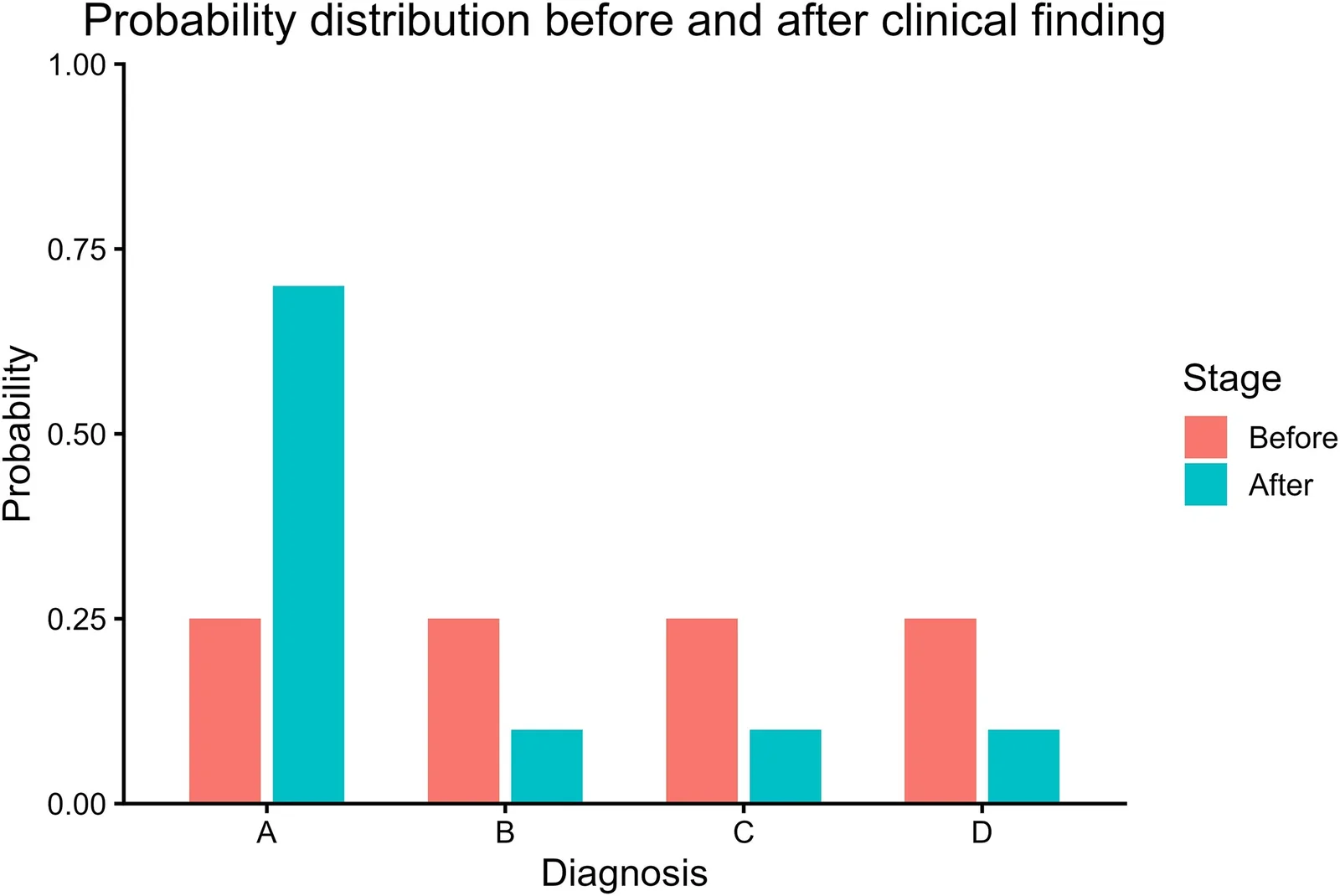
Effect of a new clinical finding on the probability distribution of diagnoses. Before the finding, diagnoses {A, B, C, D} are equally probable; afterwards, diagnosis A dominates the distribution.

The corresponding change in entropy is shown in Fig. 2. Before the finding, *H* = 2.0 bits; afterwards, *H* = 1.36 bits. The difference (0.64 bits) is the *information gain*.

**Figure 2:**
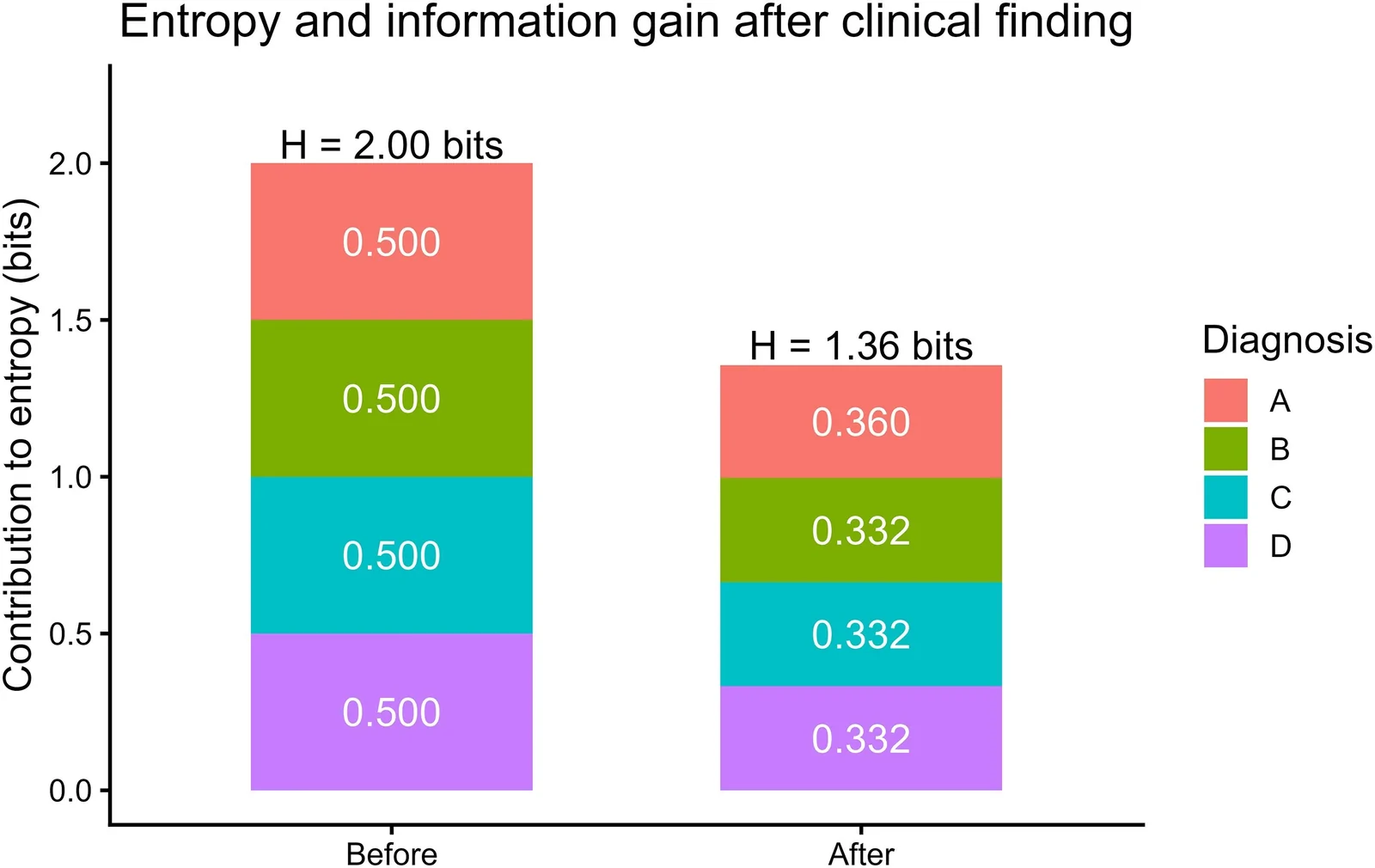
Entropy before and after the clinical finding. Entropy is the sum of the individual contributions (shown in white), corresponding to the height of each column. The reduction in entropy (0.64 bits) is the information gain.

$H_{after}$ is closer to zero, and so the GP gains a lot of information from this feature and is more certain about the diagnosis. Thus, the more information a test result carries, the more this reduces the uncertainty on the most likely diagnosis. The strength of the evidence is reflected in the magnitude of this change: findings that produce larger shifts in the probability distribution yield greater information gain than those that alter probabilities only marginally. For example, in probabilistic terms, a test that shifts the probability of a diagnosis from 10% to 70% carries far more information than one that shifts the probability from 10% to 13%, even if both tests are technically “positive.” This distinction is intuitive to clinicians, but information theory provides an explicit framework for describing and quantifying it. Making these principles explicit facilitates teaching, critical reflection, and the rational selection of questions and investigations.

There are two important consequences of this. The first is that information gain is cumulative, so that multiple tests, which individually may provide little information, when combined can substantially decrease the uncertainty. This mirrors the sequential nature of diagnostic reasoning, where clinicians begin with taking a history, examining the patient, and then ordering more invasive tests in order to gain as much information on the possible diagnoses.

The second is that information gain provides a quantitative framework for deciding which test to apply next to the patient or how to order the tests so we may optimally converge on the most likely diagnosis. In primary care, where over-testing carries costs for both the patient and the health service, information gain provides a principled framework for selecting and sequencing investigations.

These concepts allow us to frame diagnostic reasoning as a structured scientific process of sequentially reducing uncertainty so we may converge on a working diagnosis that is supported by the available evidence.

## How does information theory relate to other diagnostic frameworks?

Information theory integrates well with many existing models of diagnostic reasoning, providing a framework of concepts that clinicians already use intuitively. How it relates to three approaches used in diagnosis is discussed below.

### Bayesian reasoning

Under a Bayesian approach, diagnostic reasoning may be considered as a process in which the belief in an initial hypothesis is updated in the light of further evidence [10]. Probability is used to quantify this belief, where clinicians update the *prior* probability of a possible diagnosis with evidence from test results to form a *posterior* probability of the diagnosis using Bayes’ theorem [11]. Thus, Bayesian reasoning quantifies how much new information modifies probability estimates. In contrast, information theory quantifies how much new information reduces our uncertainty in the distribution of possible diagnoses. Information theory therefore adds a deeper layer to Bayesian decision-making by quantifying the value of information, not just its directional effect.

### Hypothetico-deductive reasoning

In the hypothetico-deductive model, the clinician generates hypotheses, tests them against new data, and discards or refines them accordingly [12] and there is some empirical evidence that such reasoning is used by some clinicians in complex cases [8, 13]. In this context, information theory helps quantify which tests or questions are most valuable in challenging or confirming a hypothesis. A hypothesis that survives several high-information challenges becomes robust; one that collapses after a single discriminating finding is one that is not well supported.

### Heuristics and intuition

Most of diagnostic reasoning in primary care is intuitive. Experienced GPs often recognize disease patterns before consciously analysing the features [7, 8, 14]. Information theory helps explain why some of these intuitive judgments are reliable.

Clinicians develop diagnostic heuristics through repeated exposure to patterns of information gain. They learn which findings most reliably reduce uncertainty. In this sense, information theory provides a useful framework for understanding intuitive diagnostic reasoning.

## Case examples

### Case 1: chest pain in primary care—stepwise reduction in uncertainty

A 55-year-old man presents after having had an episode of central chest discomfort that began while gardening. At first, the differential diagnosis is broad: musculoskeletal pain, gastro-oesophageal reflux, angina, anxiety, and less commonly, oesophageal spasm or pulmonary embolism. The diagnostic entropy is high.

#### The history

The discomfort is exertional and relieved by rest. This single feature dramatically shifts the distribution of probabilities. Musculoskeletal pain and reflux are now less plausible, whereas the uncertainty around angina as a possible diagnosis has fallen. Exertional chest pain has high information content in this context.

We learn that he has type 2 diabetes and a strong family history of ischaemic heart disease. This information again shifts the probabilities, although less dramatically than the exertional nature of the pain.

#### Examination

The patient is pain-free at rest, with normal vital signs. This provides relatively little new information and the entropy falls only slightly.

A resting ECG performed in practice is normal. The reduction in uncertainty is modest; the absence of changes does not exclude angina. The information gain remains low.

#### Investigation

A raised troponin substantially decreases entropy. A diagnosis that was merely probable now becomes overwhelmingly likely.

This stepwise sequence illustrates how different pieces of information vary in their diagnostic value. It also demonstrates that the same test (e.g. an ECG) can have very different information gains depending on the prior distribution of probabilities.

### Case 2: dysphagia-why rare features are often the most informative

A 72-year-old woman presents with difficulty swallowing solids. The initial differential includes benign oesophageal stricture, gastro-oesophageal reflux with oesophagitis, motility disorders, and malignancy.

#### Early information: high entropy

Dysphagia alone produces a moderately wide range of possibilities. The uncertainty is substantial.

#### The first discriminating feature

She reports unintentional weight loss and progressive worsening over weeks. These two features are relatively uncommon among benign causes and carry high information content. They shift the differential sharply towards malignancy.

#### Examination

A palpable left supraclavicular node provides a rare but highly informative sign. Diagnostic entropy collapses; although further tests (endoscopy, biopsy, imaging) are needed for confirmation and staging, the diagnostic reasoning in primary care is essentially complete. This case demonstrates how a single rare but discriminating feature can dramatically reduce diagnostic uncertainty.

### Case 3: rash and fever in a child—importance of uncommon early findings

A 4-year-old child presents with fever, irritability, and a “viral” rash. At first glance, this appears to be a common presentation with high entropy: viral illnesses, scarlet fever, urinary infections, and early meningococcal disease are all possible.

#### Early consultation

At this stage, all common diagnoses are plausible. The diagnostic distribution is flat; i.e. no single diagnosis has emerged as dominant.

#### The unexpected feature

On closer examination, the clinician notices mottled skin and delayed capillary refill in the lower limbs. These findings are concerning for circulatory compromise and substantially alter the diagnostic landscape. They make uncomplicated viral illness less likely and increase consideration of serious bacterial infection (such as meningococcal disease [15]) and other causes of significant circulatory compromise. Importantly, the findings do not simply shift probabilities within the existing differential; they may also prompt the clinician to expand the differential to include diagnoses that had not previously been considered.

#### Impact on decision-making

The information gained from these subtle signs, in terms of guiding the next clinical action, is far greater than that from ordering additional routine tests. It triggers immediate escalation: hospital admission, empirical antibiotics, and safety-netting (clear advice on when and how to seek further medical assessment if the patient's condition changes).

This case highlights the importance of revising diagnostic frameworks as knowledge evolves. Features once thought irrelevant may become highly informative as our understanding improves.

### Case 4: chronic cough—multiple low-yield tests without reducing uncertainty

A 42-year-old nonsmoker presents with a 6-month history of dry cough. She has no systemic symptoms, her examination is normal, and her past medical history is unremarkable.

#### Early phase: persistently high entropy

The initial differential is broad: gastro-oesophageal reflux, asthma, postnasal drip, ACE inhibitor use, chronic infection, bronchiectasis, and, less commonly, malignancy. Several low-yield investigations are performed over a period of weeks: a normal chest X-ray, routine blood tests, a peak flow diary, and empirical trials of proton pump inhibitors and intranasal steroids. None of these significantly shift the diagnostic probabilities. The entropy remains high.

#### The high-yield finding

When asked specifically about symptom variability, the patient mentions that the cough worsens after exercise and exposure to cold air, features she had not previously reported because she had not associated them with her cough. These details, though subtle, considerably increase the likelihood of asthma. A single spirometry with reversibility testing confirms the diagnosis.

#### Impact

This example illustrates the inefficiency of accumulating multiple low-information tests. A targeted history focusing on high-yield discriminating features would have provided far greater information gain early in the process

## Discussion

As a first point of access to healthcare, primary care provides an opportunity to diagnose and treat patients early in the disease process [16]. However, in many cases the presentation has vague or nonspecific features before clearer diagnostic patterns have had time to emerge. Even when patients present with more developed symptoms, these may be common to many illnesses or suggest conditions for which definitive tests are not readily available. The diagnostic process is therefore fraught with uncertainty, even for experienced GPs.

Importantly, how the GP navigates this uncertainty can, in some cases, have life-changing consequences for the patient. Diagnostic error remains a significant contributor to patient harm, with delayed or missed diagnoses carrying substantial clinical, emotional and financial consequences [17–19]. The burden is shared by clinicians, who may experience stress and frustration when an adverse outcome arises despite sincere and attentive practice [19].

Thus, the need for a systematic method when dealing with complex cases in primary care is imperative if the risk of diagnostic and clinical error is to be mitigated. On this basis, information theory offers a structured framework for analysing and responding to diagnostic uncertainty [4–6].

Principally, information theory offers insight into how diagnostic uncertainty shifts as new information is gathered. It enables clinicians to recognize when uncertainty remains high and when more discriminating data are required to guide decision-making.

It also clarifies why rare findings often carry the greatest diagnostic weight. Common symptoms may contribute little to reducing uncertainty, whereas a single unusual feature can sharply reshape the differential diagnosis. Understanding this distinction helps clinicians prioritize the elements of the history and examination that are most likely to be informative.

In addition, information theory encourages a more thoughtful use of investigations.

Tests can be evaluated not only in terms of their sensitivity and specificity, but also by the degree to which they are expected to reduce uncertainty in a particular clinical context. This perspective helps avoid superficial reassurance from low-information tests and promotes more efficient, patient-centred diagnostic strategies.

But just as the philosophy of science cautions against the uncritical use of deductive or inductive reasoning [20], information theory also comes with important limitations when applied in the diagnostic context. In clinical practice, probabilities are not always knowable with precision, as prevalence varies across settings, patient groups, and patterns of presentation [21]. Moreover, information theory does not determine management decisions; a test that offers substantial informational value may still be unsuitable if it is invasive, expensive, or disproportionate to the clinical context.

It also assumes that clinicians update their judgements rationally, yet real-world decision-making is inevitably shaped by cognitive biases [22, 23], heuristics, time pressure, and the surrounding clinical environment. Furthermore, much of the evidence underpinning diagnostic probabilities comes from secondary care, where patient populations and disease prevalence differ significantly from those seen in general practice, limiting the direct applicability of such data.

A further consideration is that the diagnostic sample space may not be fully specified at the beginning of the diagnostic process and evolve throughout the consultation. New findings may alter the probability of existing diagnoses or reveal previously unconsidered possibilities. Information theory can describe the resulting probability distribution, but generating and revising the diagnostic sample space remains dependent on clinical knowledge and reasoning.

Whilst these constraints do not diminish the usefulness of information theory, they serve as a reminder that it should be applied thoughtfully and with awareness of the context in which clinical decisions are made. In this regard, it should not be seen as a replacement for Bayesian reasoning, heuristics, or clinical experience but as a framework that complements these traditions by offering a structured way to understand how diagnostic uncertainty changes as additional clinical detail becomes available.

As with other forms of scientific reasoning, information theory encourages explicit thinking about our assumptions, our hypotheses, and our evidence. It reminds us that diagnosis is not merely the act of naming a condition but the scientific process of navigating uncertainty. By recognizing and quantifying uncertainty, information theory enhances both the safety and the rationality of diagnostic practice. In the complex and unpredictable environment of primary care, this perspective may offer a useful way of understanding and navigating diagnostic uncertainty.

## Data Availability

No new data were generated or analysed in support of this research.

## Appendix: Entropy, information content and information gain

*1. Entropy*

Suppose there are *n* possible diagnoses with probability distribution, $P=(\;p_{1},p_{2},\ldots ,p_{n})$

The *entropy*, *H*, measured in bits, is defined as

$$H=\;-\overset{n}{\underset{i}{\sum }}\;p_{i}\mathrm{lo}g_{2}(p_{i})$$

So in our earlier example where there were 4 possible diagnoses which were equally likely and *P* = *(0.25, 0.25, 0.25, 0.25),* the entropy, *H*

$$\begin{array}{rl} H & =-[0.25\log_{2}(0.25)+\;0.25\log_{2}(0.25)\\ & \;+0.25\log_{2}(0.25)+0.25\log_{2}(0.25)] \end{array}$$

H=2.00bits

*2. Information content*

If a clinical finding has probability *P* before it is observed, its *information content*, *I*, also measured in bits, is given by

$$I=\;-\log_{2}\;(P)\;bits$$

Thus a rare feature that has P=1% carries $-\log_{2}\;(0.01)$ = 6.6 bits of information

*3. Information gain*
  $$Information\;gain=\;H_{before}-H_{after}$$

In our example, the new entropy after the GP elicits a clinical feature is given by

$$H_{after}=\;-[0.7\log_{2}(0.7)+\;0.1\log_{2}(0.1)+0.1\log_{2}(0.1)+0.1\log_{2}(0.1)]$$

$$H_{after}=1.356\approx 1.36\;bits$$

Thus, $Information\;gain=\;H_{before}-H_{after}=2.00-1.36=0.64\;bits$.
